# Epithelial to Mesenchymal Transition Transcriptional Regulator ZEB1 in Liver Cancer: Oncogenic Roles and Therapeutic Potential

**DOI:** 10.3390/ijms262211135

**Published:** 2025-11-18

**Authors:** Ester Gonzalez-Sanchez, Carlos Andres Roldan-Hernandez, Ana Martin-Ramirez, Lucia Garcia-Collado, Laura Fouassier, Javier Vaquero

**Affiliations:** 1 HepatoBiliary Tumours Lab, Centro de Investigación del Cáncer and Instituto de Biología Molecular y Celular del Cáncer, CSIC-Universidad de Salamanca, 37007 Salamanca, Spain; e.gonzalezsan@usal.es (E.G.-S.); croldanh@usal.es (C.A.R.-H.); anamr02@usal.es (A.M.-R.); luciiiacollado@usal.es (L.G.-C.); 2 Department of Physiology and Pharmacology, University of Salamanca, 37007 Salamanca, Spain; 3 National Biomedical Research Institute on Liver and Gastrointestinal Diseases (CIBERehd), Instituto de Salud Carlos III, 28029 Madrid, Spain; 4 Université Paris Cité, NABI, CNRS UMR8175, Inserm U1334, F-75006 Paris, France; laura.fouassier@inserm.fr

**Keywords:** ZEB1, hepatocellular carcinoma, cholangiocarcinoma, EMT

## Abstract

Zinc finger E-box binding homeobox 1 (ZEB1) is a member of the zinc finger homeodomain transcription factor family, with a pivotal role in regulating the epithelial to mesenchymal transition (EMT) process. Increasing evidence suggests that ZEB1 is overexpressed in liver tumors, including hepatocellular carcinoma (HCC) and cholangiocarcinoma (CCA), and it correlates with advanced disease features and reduced overall survival. Here, we examine ZEB1 molecular functions, regulatory networks and contribution to tumorigenesis. We also discuss the emerging therapeutic strategies and future research directions aimed at targeting the ZEB1 molecular network to improve the outcome of liver cancer patients.

## 1. Introduction

Liver cancer includes hepatocellular carcinoma (HCC), which is the most prevalent form of primary liver cancer and accounts for approximately 75–85% of cases [1], and cholangiocarcinoma (CCA), which arises from the epithelial cells that line the bile ducts, and represents around 10–15% of all liver cancer cases [2]. Despite fundamental differences in etiological factors, genetic drivers and tumor microenvironment (TME) characteristics, both HCC and CCA are characterized by aggressive behavior, poor prognosis and limited therapeutic options, especially in the advanced stages of the disease, which occur in the majority of patients due to frequent late-stage diagnosis of these malignancies [1,2]. Consequently, liver cancer currently ranks as the third leading cause of cancer-related mortality [3].

Among the mechanisms leading to cell dissemination and metastasis of cancer cells, the epithelial to mesenchymal transition (EMT) is probably the most widely studied mechanism in this regard [4]. EMT, originally observed during embryogenesis, is a reversible dynamic process during which epithelial cells gradually adopt the structural and functional characteristics of mesenchymal cells, including motility, invasiveness, stemness and chemoresistance [4,5]. This process is tightly regulated by different environmental signals from major signaling pathways (i.e., transforming growth factor-beta (TGF-β), Fibroblast growth factor (FGF), wingless/integrated (Wnt), etc.) and a group of EMT-inducing transcription factors (EMT-TF) belonging to three main families (zinc finger E-box-binding homeobox 1/2 (ZEB1/2), snail family transcriptional repressor 1/2 (SNAI1/2) and twist family bHLH transcription factor 1/2 (TWIST1/2)) [4,5]. These factors, mainly identified as negative regulators of epithelial markers such as E-cadherin, have been involved in a plethora of molecular mechanisms involved in various biological processes since then [6,7,8].

The ZEB (Zinc finger E-box-binding homeobox) protein family of transcription factors was first discovered in *Drosophila melanogaster* and is constituted by two members, ZEB1 and ZEB2 [9]. Both of them share structural similarities and contain two zinc finger clusters that allow them to bind DNA regulatory sequences and, therefore, control the expression of their targets through promoter binding [9]. ZEB1 and ZEB2 are known for their ability to stimulate EMT, but some reports indicate that they can have both redundant and opposing functions, even in the same type of cancer [9,10,11].

The most studied ZEB protein is undoubtedly ZEB1, which has been shown to play multiple roles in regulating the aggressivity and chemoresistance of different tumor types [12]. Emerging evidence suggests that ZEB1 is overexpressed in liver tumors compared to normal liver tissue, and that its expression correlates with advanced disease features such as vascular invasion, intrahepatic metastasis and reduced overall survival [13,14]. Thus, understanding ZEB1-related functions in liver cancer may introduce new ways to therapeutically challenge these aggressive malignancies.

In this review, we aim to give a comprehensive overview of the current knowledge on the biological roles of ZEB1 in the two main subtypes of liver cancer, HCC and CCA. We will first introduce the structural and functional characteristics of ZEB1, followed by a detailed examination of its role in tumor initiation, progression and resistance mechanisms in both types of liver cancer. Finally, we will discuss emerging therapeutic strategies and future directions for research, aimed at exploiting ZEB1 as a biomarker and therapeutic target.

## 2. ZEB1 Structural Features and Functional Domains

**Figure 1:**
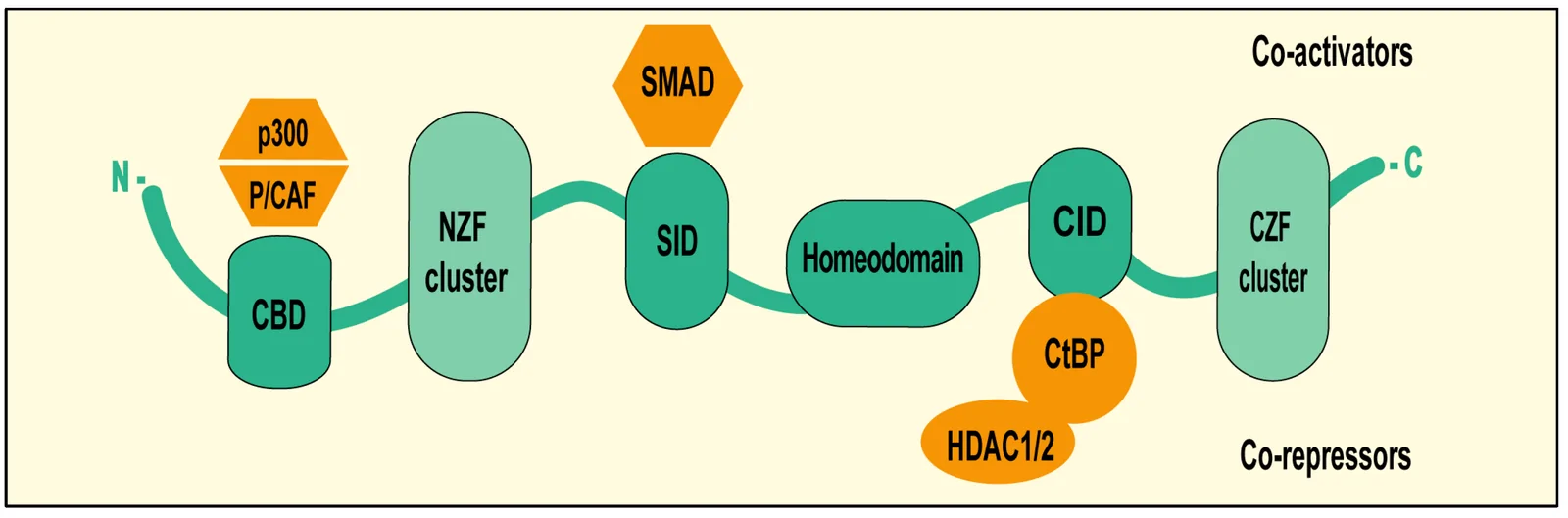
Schematic structure of ZEB1. ZEB1 is formed by two DNA-binding zinc finger domains located near the N-terminal (NZF) and C-terminal (CZF) regions, a central homeodomain, binding sites for SMADs (SID), CtBP (CID) and the p300-P/CBP-associated factor binding domain (CBD).

The ZEB1 protein (also known as TCF8 and δEF1) is encoded by the homonym gene located on chromosome 10p11.22 [15]. The protein consists of 1117 amino acids and is a transcription factor that features two clusters of C2H2-type zinc finger domains at both the N-terminal and C-terminal regions, which are responsible for binding to the E-box sequences in target gene promoters with a consensus sequence of 5′-CANNTG-3′ (Figure 1) [16]. The middle region contains a homeodomain that contributes to protein–protein interactions. In addition, ZEB1 contains a Smad interaction domain (SID), a CtBP interaction domain (CID) and p300-P/CBP-associated factor (P/CAF) binding domain (CBD) that are key in the control of its transcriptional activity (Figure 1) [17]. ZEB1 may upregulate or downregulate target gene transcription through the recruitment of different coactivators or cosuppressors via SID, CID and CBD [17]. For example, ZEB1 can directly interact with SMADs through SID to regulate TGF-β-responsive genes. Similarly, p300 and P/CAF can be recruited to CBD to activate gene transcription. Conversely, ZEB1 can repress target gene transcription through the recruitment of the CtBP corepressor complex (histone deacetylases HDAC1/2) via CID (Figure 1) [18]. These cofactors allow ZEB1 to modulate transcription with epigenetic mechanisms, including DNA methylation or histone modifications [19,20]. This extensive list of partners allows ZEB1 to play pleiotropic functions in various biological processes, from embryogenesis to cancer.

## 3. Regulation of ZEB1 Expression in Liver Cancer

### 3.1. Transcriptional Regulation

**Figure 2:**
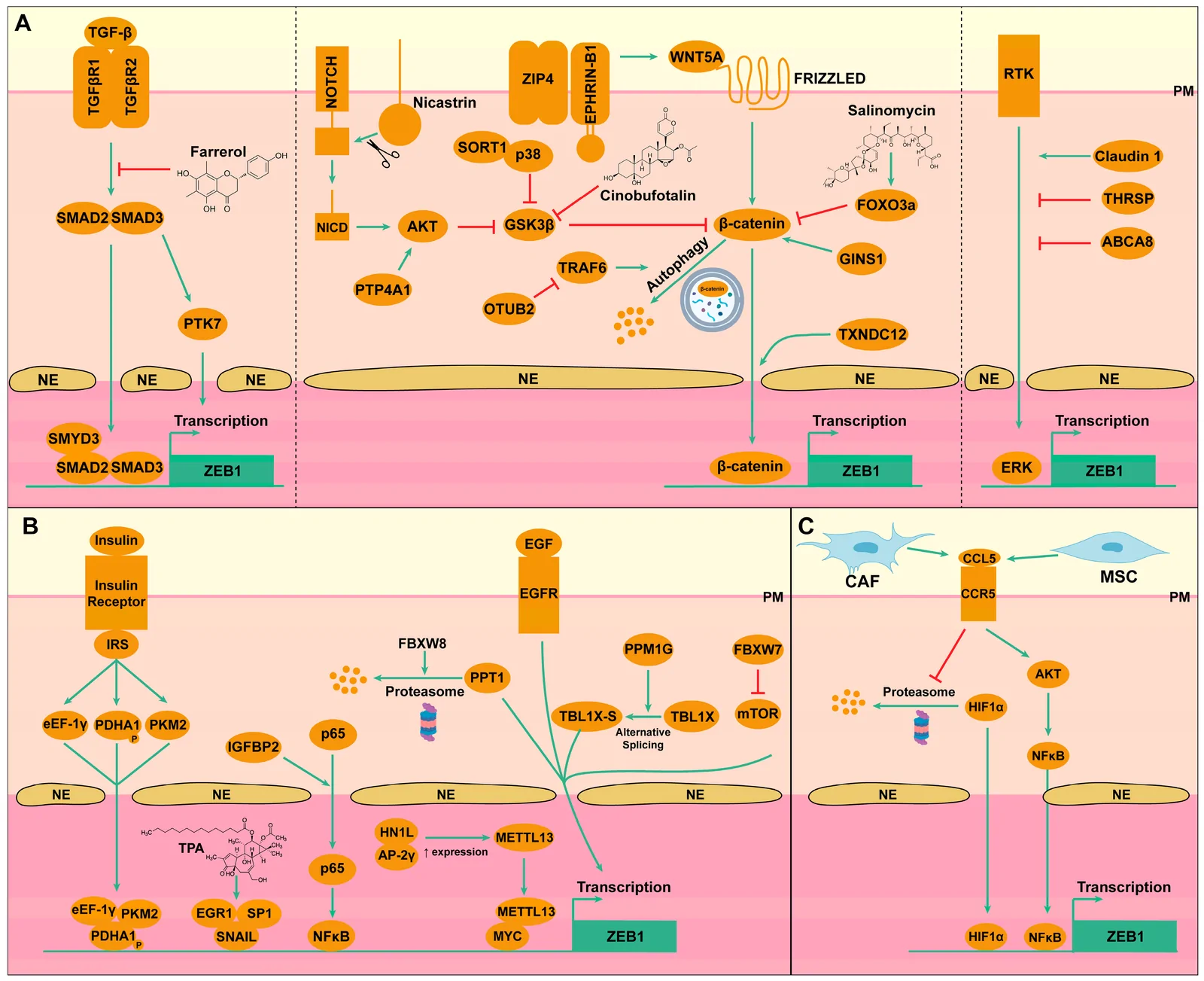
Mechanisms of transcriptional regulation of *ZEB1* expression in liver cancer. The different mechanisms involved in the regulation of the *ZEB1* promoter are depicted in this figure and detailed in the text. (**A**) Main signaling pathways regulating *ZEB1* transcription. (**B**) *ZEB1* transcriptional regulation by other pathways. (**C**) Impact of tumor microenvironment in *ZEB1* transcriptional regulation. ABCA8, ATP binding cassette subfamily A member 8; AKT, AKT serine/threonine kinase; AP-2, Activator Protein-2 gamma; CCL5, C-C motif chemokine ligand 5; CCR5, C-C motif chemokine ligand 5 receptor; eEF-1γ, eukaryotic translation elongation factor 1 gamma; EGF, epidermal growth factor; EGFR, epidermal growth factor receptor; EGR1, early growth response 1; ERK, extracellular signal-regulated kinase; FBXW7, F-box and WD repeat domain containing 7; FOXO3A, forkhead box O3; GINS1, GINS complex subunit 1; GSK3β, glycogen synthase kinase 3 beta; HF1α, hypoxia inducible factor 1 subunit alpha; HN1L, hematological and neurological expressed 1-like; IGFBP2, Insulin like growth factor binding protein 2; IRS, insulin receptor substrate; METTL13, methyltransferase 13; mTOR, mechanistic target of rapamycin kinase; NFκB, nuclear factor kappa B; NICD, notch intracellular domain; NOTCH, Neurogenic locus notch homolog protein; OTUB2, OTU deubiquitinase, ubiquitin aldehyde binding 2; PDHA1, pyruvate dehydrogenase E1 subunit alpha 1; PKM2, pyruvate kinase M2; PPM1G, Protein phosphatase 1G; PPT1, palmitoyl-protein thioesterase 1; PTK7, Tyrosine-protein kinase-like 7; PTP4A1, Protein Tyrosine Phosphatase Type 4A1; RTK, receptor tyrosine kinase; SMYD3, SET and MYND domain containing 3; SNAIL, snail family transcriptional repressor 1; SORT1, Sortilin 1; SP1, Specificity protein 1; TBL1X, transducin beta like 1 X-linked; TGF-β, transforming growth factor-beta; TGFβR1, transforming growth factor-beta receptor 1; THRSP, thyroid hormone responsive protein; TXNDC12, thioredoxin domain containing 12; TRAF6, TNF receptor associated factor 6; ZEB1, Zinc finger E-box-binding homeobox 1; ZIP4, Zrt- and Irt-like protein 4.

ZEB1 is not only a key effector of EMT but is also integrated into complex regulatory networks involving major signaling pathways, which are frequently dysregulated in liver cancers. The mechanisms of regulation of ZEB1 transcription are summarized in Figure 2. For example, TGF-β is able to upregulate ZEB1 expression in HCC cells in a SMAD2/3-dependent mechanism (Figure 2A) [21,22]. On one side, SMAD2/3 are able to upregulate the expression of Tyrosine-protein kinase-like 7 (PTK7), which in turn upregulates ZEB1 expression. On the other side SMAD2/3 are able to directly bind the ZEB1 promoter to promote ZEB1 transcription [21]. Interestingly, SET (suppressor of variegation, enhancer of zeste, trithorax) and MYND (Myeloid-Nervy-DEAF1) domain containing 3 (SMYD3) is required for TGF-β-induced EMT in HCC cells that are resistant to sorafenib. Indeed, SMYD3 interacts with SMAD2/3 and epigenetically promotes the expression of ZEB1 [21].

Another major signaling cascade involved in *ZEB1* regulation is the Wnt/β-catenin pathway (Figure 2A). Generally, WNT signaling activation disrupts the binding of Glycogen synthase kinase 3 beta (GSK3β) to β-catenin to allow the later to translocate to the nucleus and activate target gene transduction, including ZEB1, but several other molecules can modulate this cascade in liver cancer. For example, upstream WNT, Zrt- and Irt-like protein 4 (ZIP4), a zinc transporter located at the plasma membrane, inhibits by direct binding the ubiquitination of Ephrin-B1, which in turn activates WNT5A signaling in HCC cells [23]. Downstream of WNT, several mechanisms also regulate the activity of β-catenin in HCC cells. A thioredoxin domain containing 12 (TXNDC12) interacts with β-catenin and stimulates the nuclear translocation and activation of β-catenin [24]. Nicastrin (NCSTN) also facilitates the translocation of β-catenin to the nucleus. More precisely, Nicastrin activates Notch1 cleavage, which in turn activates AKT serine/threonine kinase (AKT) signaling, leading to GSK3β phosphorylation and liberation of β-catenin from the GSK3β/β-catenin complex [25]. Similarly, Sortilin 1 (SORT1) is able to bind p38 to enhance its stability and promote GSK-3β phosphorylation and the liberation of β-catenin [26]. Other factors affect β-catenin signaling in HCC cells. GINS complex subunit 1 (GINS1) promotes EMT and tumor metastasis by favoring β-catenin signaling [27], while Forkhead box O3 (FOXO3a) reduces the binding of β-catenin to the T-cell factor (TCF) and inhibits β-catenin/TCF target gene expression [28]. Although less studied, some other mechanisms regulating β-catenin have been identified in CCA. For example, the ubiquitin thioesterase OTU deubiquitinase, ubiquitin aldehyde binding 2 (OTUB2), is able to inhibit the lysosomal degradation of β-catenin by interacting with the TNF receptor-associated factor 6 (TRAF6), resulting in increased expression and signaling of β-catenin [29]. Another mechanism involves Protein tyrosine phosphatase type 4A1 (PTP4A1), which activates AKT signaling, which in turn phosphorylates GSK3β, liberating β-catenin to translocate to the nucleus and activate its target gene transcription [30].

Another ZEB1 major regulatory cascade is the Mitogen-activated protein kinase (MAPK) signaling pathway (Figure 2A). Activation of different receptors leads to the activation of this pathway, which ends with the translocation of phosphorylated extracellular signal-regulated kinase (ERK) to the nucleus and its binding to the ZEB1 promoter. In HCC cells, the thyroid hormone responsive protein (THRSP) and ATP binding cassette subfamily A member 8 (ABCA8) inhibit ERK signaling and ZEB1-induced EMT [31,32]. On the contrary, claudin-1 activates the MAPK pathway and ZEB1 expression [33].

Other signals can also regulate ZEB1 expression (Figure 2B). In HCC cells, insulin binding to the insulin receptor triggers the phosphorylation of pyruvate dehydrogenase E1 subunit alpha 1 (PDHA-1) and its nuclear translocation in the company of eukaryotic translation elongation factor 1 gamma (eEF-1γ) and pyruvate kinase M2 (PKM2), which together bind to the ZEB1 promoter to activate its expression [34]. Insulin-like growth factor binding protein 2 (IGFBP2), another protein from the insulin family, mediates the nuclear localization of p65 in HCC cells. p65 is then able to activate nuclear factor kappa B (NFκB) to promote ZEB1 transcription [35]. Hematological and neurological expressed 1-like (HN1L) acts in concert with Activator Protein-2 gamma (AP-2γ) to promote the transcription of methyltransferase 13, (METTL13), which then cooperates with MYC to transcriptionally upregulate the expression of ZEB1 in HCC cells [36]. Also in HCC cells, Protein phosphatase 1G (PPM1G) alters the alternative splicing of transducin beta like 1 X-linked (TBL1X) favoring the TBL1X-S isoform, which acts as an oncogene by promoting ZEB1 expression [37]. Other EMT-TF, such as Snail, can regulate ZEB1 expression. Indeed, exposure of HCC cells to TPA (O-tetradecanoyl-phorbol 13-acetate) promotes Snail binding to the ZEB1 promoter in a complex involving early growth response 1 (EGR1) and Specificity protein 1 (SP1) [38]. On the contrary, F-box and WD repeat domain containing 8 (FBXW8) acts as a negative regulator of ZEB1 expression by promoting the proteasomal degradation of Palmitoyl-Protein Thioesterase 1 (PPT1), which is an activator of ZEB1 expression in HCC cells [39]. Another F-box protein, F-Box and WD Repeat Domain Containing 7 (FBXW7) negatively regulates ZEB1 expression by inhibiting the mechanistic target of rapamycin kinase (mTOR) signaling in CCA cells [40].

Most of these regulatory mechanisms involve autocrine loops that activate signaling cascades in HCC or CCA cells, but signals coming from the TME can also regulate ZEB1 expression (Figure 2C). In this regard, C-C motif chemokine ligand 5 (CCL5) regulates ZEB1 in liver cancer. In HCC, cancer-associated fibroblasts (CAF) produce CCL5 that, upon binding to tumor cells, activates signals that interfere with the ubiquitination and degradation by the proteasome of hypoxia-inducible factor 1 alpha (HIF1α). Then, HIF1α translocates to the nucleus and binds to the ZEB1 promoter to increase its expression [41]. In CCA, mesenchymal stem cells (MSC) are also able to produce CCL5 upon exposure to pro-inflammatory signals. CCL5 then activates the expression of ZEB1 through a mechanism involving AKT-NFκB signaling activation.

On top of these signaling cascades, different environmental cues have been shown to regulate the expression of ZEB1. Indeed, some etiologic factors that lead to the development of HCC, such as alcohol, hepatitis virus (HCV and HBV) or high-fat diet consumption, can induce ZEB1 expression [42,43,44,45]. In the case of ethanol, the mechanism has been associated with the induction of long non-coding (lnc) RNA lnc171, which in turn sponges microRNA (miRNA or miR) mir-873-5p, a negative regulator of ZEB1 mRNA expression [43]. Ethanol and hepatitis virus have been linked to ZEB1 through a mechanism involving chromosome 8q24 amplification, which leads to MYC expression, that in turn promotes ZEB1 expression [42]. In the case of a high-fat diet, prolonged exposure to fatty acids increased the levels of TGF-β and β-catenin, important EMT inducers and ZEB1 regulators [44]. Nevertheless, other environmental factors besides etiologic factors can regulate ZEB1. Di(2-ethylhexyl) phthalate (DEHP), a worldwide common plasticizer, is often observed in patients because of the continual usage of plastic medical devices. Interestingly, long-term DEHP exposure induces EMT by increasing the expression of EMT-TF, including ZEB1 [46]. High expression of ZEB1 in response to these environmental signals increases the invasiveness, stemness and even resistance to sorafenib, of HCC cells [43,44,45,46].

### 3.2. Post-Transcriptional Regulation

**Figure 3:**
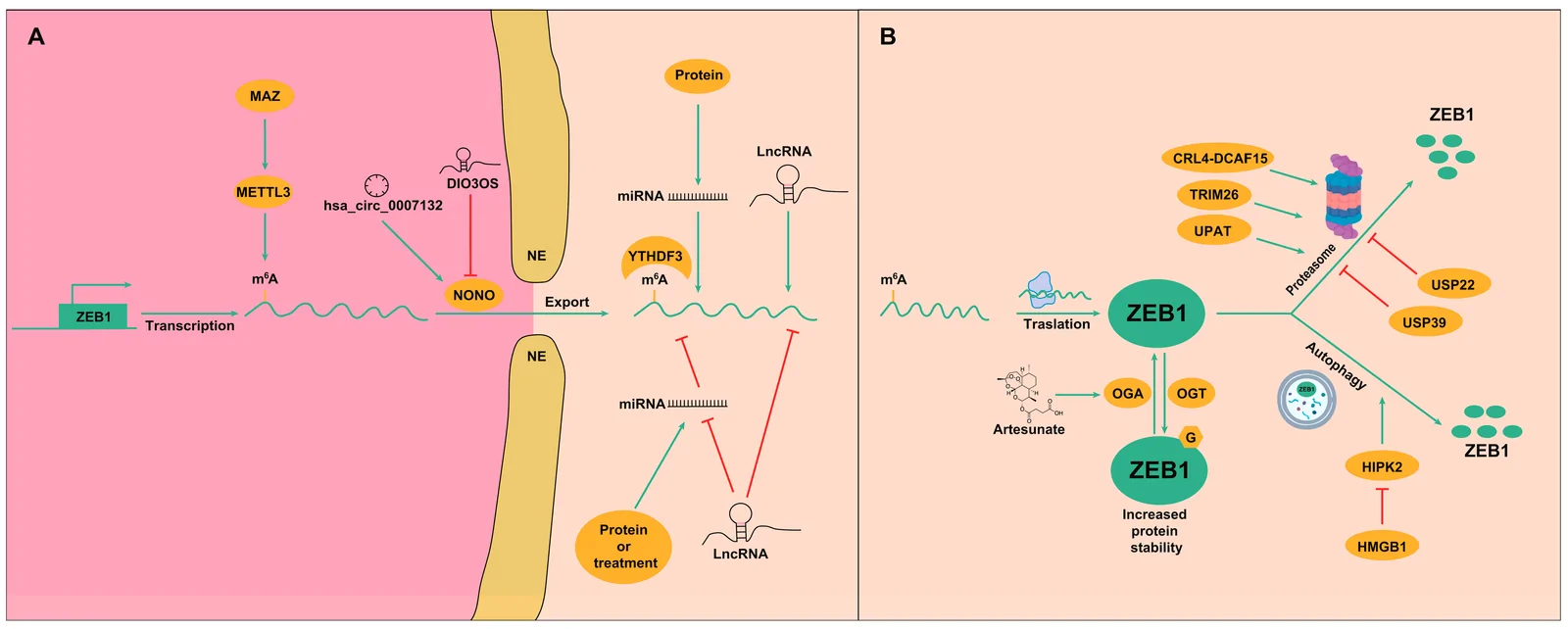
Mechanisms involved in the regulation of ZEB1 expression in liver cancer at the post-transcriptional (**A**) and post-translational (**B**) level. (**A**) Post-transcriptional mechanisms include mRNA modifications and non-coding RNA regulation. (**B**) Post-transcriptional mechanisms include protein modifications that regulate protein stability and ZEB1 degradation by the proteasome or by autophagy. circRNA, circular RNA; CRL4-DCAF15, DDB1 and CUL4 associated factor 15; HIPK2, Homeodomain Interacting Protein Kinase 2; HMGB1, high mobility group box 1; lncRNA, long non-coding RNA; MAZ, MYC associated zinc finger protein; METTL3, Methyltransferase-like protein 3; iRNA, microRNA; NONO, non-POU domain containing octamer binding; OGA, O-GlcNAcase; OGT, O-GlcNAc transferase; TRIM26, tripartite motif containing 26; UPAT, ubiquitin-like plant homeodomain (PHD) and really interesting new gene (RING) finger domain containing protein 1 (UHRF1) protein-associated transcript; USP22, ubiquitin specific peptidase 22; USP39, ubiquitin specific peptidase 39; YTHDF3, YTH domain-containing family protein 3; ZEB1, Zinc finger E-box-binding homeobox 1.

Besides the above-described regulatory signals, ZEB1 is also under tight post-transcriptional regulations at different levels, including mechanisms that regulate the mRNA export to the cytoplasm, the addition of mRNA modifications, and regulation by non-coding RNAs (ncRNAs), which are RNAs that do not encode proteins but play an important role in post-transcriptional control of gene expression. These ncRNA are classified based on length and structure into lncRNA, miRNA and circular RNA (circRNA). All these post-transcriptional mechanisms are summarized in Figure 3. In terms of RNA modifications, N6-methyladenosine (m^6^A) is the only mRNA modification identified so far in the regulation of ZEB1 in HCC. In this sense, the MYC-associated zinc finger protein (MAZ) is involved in m^6^A methylation in HCC by targeting the transcriptional regulation of key m^6^A enzymes [47]. Indeed, MAZ can upregulate the expression of the methylase Methyltransferase-like protein 3 (METTL3) by directly binding to its promoter. In turn, METTL3 can methylate its targets, among which ZEB1 has been identified in HCC. METTL3-mediated m^6^A methylation of ZEB1 mRNA increases its stabilization [47,48]. Besides writer methylases, the reader YTH domain-containing family protein 3 (YTHDF3) also regulates the stability of ZEB1 in HCC through the m^6^A modification [49]. Another layer of regulation of this process comes from circ_KIAA1429, which is overexpressed in HCC and facilitates the function of YTHDF3 on ZEB1 mRNA [49]. Thus, mechanisms favoring m^6^A methylation of ZEB1 mRNA play a role in increasing the invasion and metastasis of HCC by inducing EMT.

Regarding ncRNAs, the most widely described mechanism is the double-negative feedback loop that ZEB1 forms with the miR-200 family, wherein ZEB1 represses the transcription of miR-200 members while being targeted for degradation by the same miRNAs: a mechanism that allows fine-tuned and dynamic regulation of EMT [50]. However, multiple other miRNAs have been described to regulate ZEB1 in recent years. In addition, there are other upstream signals that may regulate the expression of miRNAs, such as lncRNA, which have gained a lot of interest, as they may regulate miRNAs by acting as sponges and, thus, reducing their availability to target ZEB1. The ZEB1 regulatory mechanisms involving non-coding RNAs are summarized in Table 1.

Apart from these mechanisms, ZEB1 can be regulated post-transcriptionally by ncRNAs without its mRNA levels being affected. A non-POU domain containing octamer binding (NONO) is an RNA-binding protein that facilitates ZEB1 mRNA nuclear export. The lncRNA DIO3OS is generally downregulated in HCC patients. This lncRNA represses ZEB1 by interacting with the NONO protein, thereby restricting NONO-mediated nuclear export of ZEB1 mRNA. Thus, reduced DIO3OS leads to increased ZEB1 protein expression, stemness and HCC progression [51]. Conversely, hsa_circ_0007132 has been found to be increased in patients following lenvatinib treatment and HCC lenvatinib-resistant cell lines. Mechanistically, hsa_circ_0007132 binds to NONO and impairs its ubiquitination-mediated degradation, thereby enhancing the NONO-mediated nuclear export of ZEB1 mRNA. Ultimately, ZEB1 increased protein levels and participates in lenvatinib resistance [52].

It is worth mentioning that while we have knowledge about various types of ncRNAs modulating ZEB1 expression in liver cancer for more than 10 years [53,54], and even longer in other tumors, reports on other posttranscriptional regulatory mechanisms such as RNA modifications (i.e., m^6^A) are as recent as 2025 [47], underscoring the novelty of a growing field in the past few years that will surely help to understand ZEB1’s role, not only in liver cancer but also many other pathological settings.

### 3.3. Post-Translational Regulation

ZEB1 protein can also undergo post-translational modifications, such as phosphorylation, ubiquitination and acetylation, which influence its stability, localization and transcriptional activity [55]. Very recently, a new form of posttranslational modification has been identified for ZEB1 in HCC, the O-GlcNAcylation. ZEB1 can be O-GlcNAcylated at ser670 by O-GlcNAc transferase (OGT), which increases the protein stability of ZEB1, while O-GlcNAcase (OGA) is able to remove the O-linked N-acetylglucosamine modification. Thus, the expression and activity of OGT and OGA may determine the protein stability of ZEB1 [56]. Nevertheless, in terms of the regulation of ZEB1 expression in liver cancer, most studies are related to ZEB1 degradation by ubiquitination mechanisms (Figure 3). Related to proteasome degradation of ZEB1, deubiquitinase ubiquitin specific peptidase 39 (USP39) and E3 ligase tripartite motif containing 26 (TRIM26) function in an antagonistic pattern in HCC, controlling ZEB1 stability to determine HCC progression. Thus, HCC with a high expression of USP39 show increased ZEB1 expression that determines higher proliferation and migration of HCC cells [57]. Similarly, ubiquitin-specific peptidase 22 (USP22) is involved in the maintenance of ZEB1 stability via its deubiquitinase activity [58]. Another factor involved in ZEB1 degradation by the proteasome is the CRL4-DCAF15 (DDB1 and CUL4 associated factor 15) E3 ubiquitin ligase complex, which recognizes the N-terminal zinc finger domain of ZEB1 to trigger its ubiquitination and posterior degradation, reducing ZEB1 expression and the proliferative and invasive abilities of HCC cells [59]. The ubiquitin-like plant homeodomain (PHD) and really interesting new gene (RING) finger domain-containing protein 1 (UHRF1) protein-associated transcript (UPAT) are also able to promote ZEB1 degradation through the ubiquitin-proteasome pathway in HCC [60]. Besides the proteasome-related mechanisms, ZEB1 protein expression can be reduced by autophagic degradation. In HCC, high mobility group box 1 (HMGB1) promotes homeodomain interacting protein kinase 2 (HIPK2) degradation. HIPK2 is a kinase that plays multifaceted roles in various cellular processes. Among them, HIPK2 regulates ZEB1’s autophagic degradation. Thus, HIPK2 degradation results in protection of ZEB1 from autophagic degradation and increases HCC cell growth. Thus, HMGB1 inhibition can suppress HCC progression via HIPK2-mediated autophagic degradation of ZEB1 [61].

**Table 1:** Mechanism of regulation of ZEB1 expression involving non-coding RNAs (miRNA and lncRNA).

Tumor	Factor	Regulates	miRNA	Regulates	Factor	Result	Ref.
HCC	Lnc-RP11-422N16.3	Negatively	miR-23b-3p	Negatively	ZEB1	↑ ZEB1	[62]
HCC	LncRNA-SNHG6	Negatively	miR-101-3p	Negatively	ZEB1	↑ ZEB1	[63]
HCC	-	-	miR-139-5p	Negatively	ZEB1	↓ ZEB1	[64]
HCC	LncRNA-TUG1	Negatively	miR-142-3p	Negatively	ZEB1	↑ ZEB1	[65]
HCC	Lnc-MALAT1	Negatively	miR-143-3p	Negatively	ZEB1	↑ ZEB1	[66]
HCC	circ-100338	Negatively	miR-143-3p	Negatively	ZEB1	↑ ZEB1	[67]
HCC	ELF3	Negatively	miR-141-3p	Negatively	ZEB1	↑ ZEB1	[68]
HCC	ATRA	Positively	miR-141-3p miR-200a-3p miR-200c-3p	Negatively	ZEB1	↓ ZEB1	[69]
HCC	LncRNA-ZFAS1	Negatively	miR-150	Negatively	ZEB1	↑ ZEB1	[70]
HCC	TGF-β	Positively	miR-155	Positively	ZEB1	↑ ZEB1	[71]
HCC	LncRNE-PE	Negatively	miR-200a miR-200b	Negatively	ZEB1	↑ ZEB1	[72]
HCC	Gα12	Negatively	miR-200a miR-200b	Negatively	ZEB1	↑ ZEB1	[73]
CCA	Lnc-ZEB1-AS1	Negatively	miR-200a	Negatively	ZEB1	↑ ZEB1	[74]
HCC	Lnc-HULC	Negatively	miR-200a-3p	Negatively	ZEB1	↑ ZEB1	[75]
HCC	Genomic deletion, promoter methylation	Negatively	miR-200b	Negatively	ZEB1	↑ ZEB1	[76]
HCC	Lnc-CARLo-5	Negatively	miR-200b	Negatively	ZEB1	↑ ZEB1	[77]
HCC	KLF4	Positively	miR-200b	Negatively	ZEB1	↓ ZEB1	[78]
HCC	YB1	Negatively	miR-200b miR-205	Negatively	ZEB1	↑ ZEB1	[79]
HCC	LncRNA-XIST	Negatively	miR-200b-3p	Negatively	ZEB1	↑ ZEB1	[80]
CCA	-	-	miR-200c	Negatively	ZEB1	↓ ZEB1	[81]
CCA	Lnc-ATB	Negatively	miR-200c	Negatively	ZEB1	↑ ZEB1	[82]
HCC	p53	Positively	miR-200	Negatively	ZEB1	↓ ZEB1	[54]
HCC	LncRNA-ATB	Negatively	miR-200	Negatively	ZEB1	↑ ZEB1	[53]
HCC	LINC00273	Negatively	miR-200	Negatively	ZEB1	↑ ZEB1	[83]
HCC	Lnc-SNHG3	Negatively	miR-326	Negatively	ZEB1	↑ ZEB1	[84]
HCC	-	-	miR-369	Negatively	ZEB1	↓ ZEB1	[85]
HCC	LncRNA-PRNCR1	Negatively	miR-411-3p	Negatively	ZEB1	↑ ZEB1	[86]
HCC	Lnc-MAPKAPK5	Negatively	miR-429	Negatively	ZEB1	↑ ZEB1	[87]
CCA	DMY	Positively	miR-455-3p	Negatively	ZEB1	↓ ZEB1	[88]
HCC	-	-	miR-590-3p	Negatively	ZEB1	↓ ZEB1	[89]
HCC	-	-	miR-708	Negatively	ZEB1	↓ ZEB1	[90]
HCC	Lnc171	Negatively	miR-873-5p	Negatively	ZEB1	↑ ZEB1	[43]
HCC	Lnc-HCCL5	-	-	Positively	ZEB1	↑ ZEB1	[91]
HCC	LncPNUTS	-	-	Positively	ZEB1	↑ ZEB1	[92]

ATRA, all-trans retinoic acid; CCA, cholangiocarcinoma; DMY, dihydromyricetin; ELF3, E74-like ETS transcription factor 3; HCC, hepatocellular carcinoma; KLF4, Kruppel-like factor 4; TGF-β, transforming growth factor β; YB1, Y-box binding protein 1. ↑ indicates increased ZEB1 expression and ↓ indicates ZEB1 reduced expression.

In addition to the above-described regulatory networks controlling ZEB1, the expression of other proteins has been linked to modified expression of ZEB1 without giving precise specifications of the regulatory mechanism. For example, increased expression of HMGB2, 14-3-3ε, POU class 2 homeobox 1 (POU2F1), fibrinogen gamma chain (FGG), RNA-binding motif, Y chromosome (RBMY), stage-specific embryonic antigen 3 (SSEA3), chromobox 6 (CBX6) has been shown to increase EMT, invasiveness and stemness of HCC cells through increasing ZEB1 expression [93,94,95,96,97,98,99], while for F-actin-capping protein subunit alpha-1 (CAPZA1), transient receptor potential cation channel subfamily V member 1 (TRPV1) and liver kinase B1 (LKB1), high expression correlates with low ZEB1 expression and reduced aggressivity of HCC cells [100,101,102]. Similarly, for adrenomedullin (ADM), cullin 4A (CUL4A) and epidermal growth factor (EGF), high expression correlates with high expression of ZEB1 and induced EMT and metastasis in CCA [103,104,105].

## 4. Role of ZEB1 in Hepatocellular Carcinoma (HCC)

Clinical evaluation showed that ZEB1 is highly expressed in malignant HCC cells compared to normal hepatocytes in 23–65.4% of the patients (Table 2) [90,106,107,108,109]. As an EMT-TF, ZEB1 expression correlated with reduced E-cadherin expression [13,106] and increased Vimentin expression [108]. Furthermore, ZEB1 expression was associated with aggressive clinical parameters, including poorly differentiated tumors, advanced TNM stage, increased number of tumors, multifocal metastases, vascular invasion (Table 3). All of these factors contributed to reduced progression-free survival and overall survival in patients with high ZEB1 expression (Table 2) [90,106,107,108,109].

**Figure 4:**
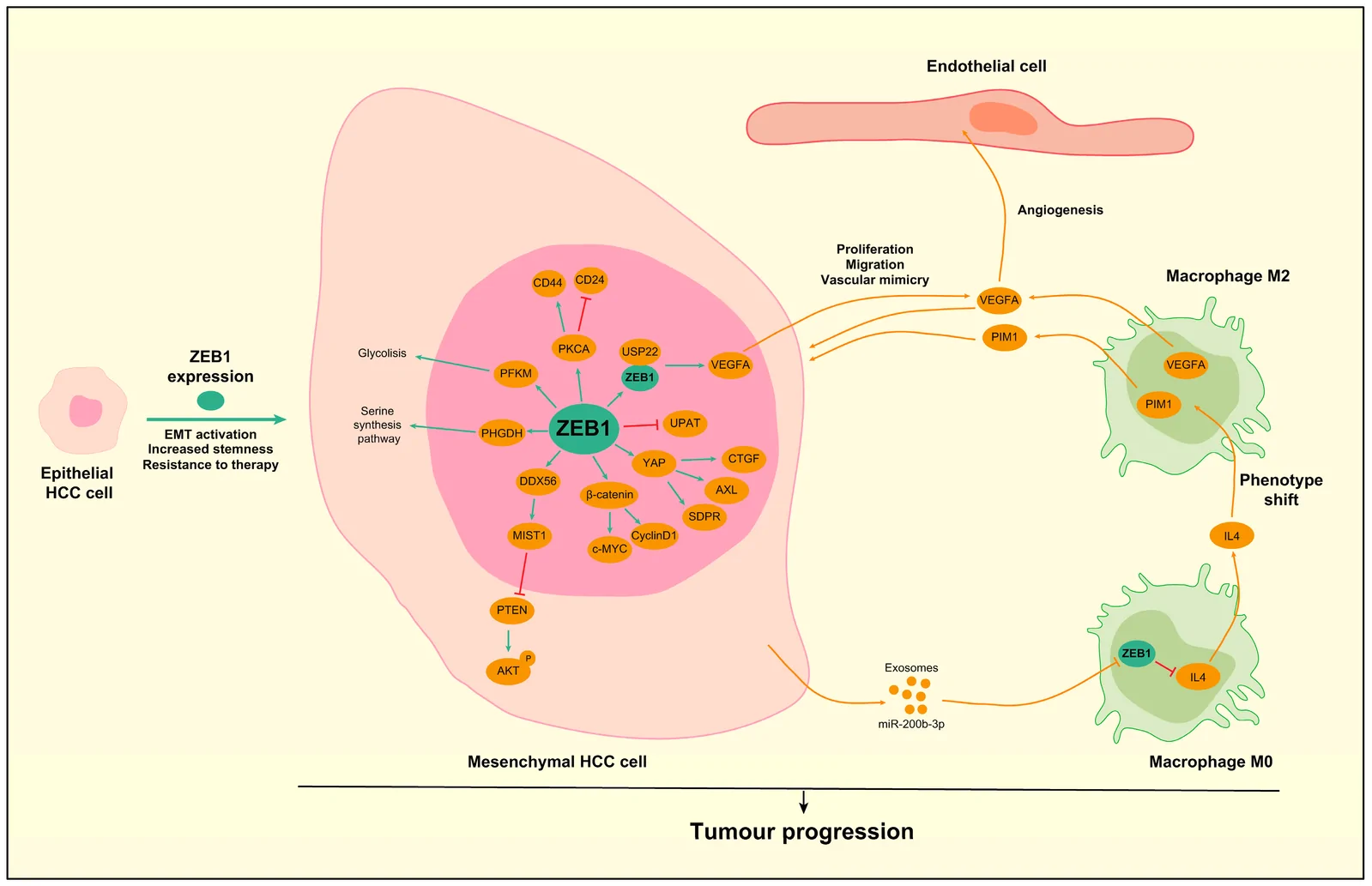
Schematic representation of the mechanisms governing HCC tumor progression and chemoresistance that are regulated by ZEB1 in tumor and tumor microenvironment cells. AKT, AKT serine/threonine kinase; CTGF, connective tissue growth factor; DDX56, DEAD-Box 56; MIST1, muscle, intestine and stomach expression 1; PFKM, phosphofructokinase-1; PHGDH, phosphoglycerate dehydrogenase; PKCA, Protein kinase C alpha; PTEN, phosphatase and tensin homolog; SDPR, serum deprivation response; UPAT, ubiquitin-like plant homeodomain (PHD) and really interesting new gene (RING) finger domain-containing protein 1 (UHRF1) protein-associated transcript; USP22, ubiquitin specific peptidase 22; VEGFA, vascular endothelial growth factor A; YAP, Yes-Associated Protein; ZEB1, Zinc finger E-box binding homeobox 1.

Preclinical in vitro studies showed that ZEB1 can promote the proliferation, colony formation, sphere formation and migration of HCC cells and markedly enhance tumor progression in vivo in diethylnitrosamine (DEN)-induced models and orthotopic xenografts of human cell lines [90,108,109,111,112]. Mechanistically, multiple factors can be regulated by ZEB1 to lead to these aggressive features, which are indicated in Figure 4. As in patient samples, in HCC cell lines, ZEB1 is able to downregulate the expression of E-cadherin and increase the expression of mesenchymal markers such as Vimentin, matrix metallopeptidase 2 (MMP2) and MMP9, contributing to the migration and invasion of these cells [90,108,109]. ZEB1 is also able to activate the Wnt/β-catenin signaling pathway by upregulating the protein expression levels of β-catenin and its downstream targets c-Myc and Cyclin D1 [90]. Interestingly, ZEB1 has been shown to interact with Yes-associated protein (YAP) to become a transcriptional activator in some aggressive cancers [113]. However, in HCC cells, ZEB1 is able to directly increase the expression of YAP and activate YAP downstream signaling with the upregulation of genes like AXL receptor tyrosine kinase (AXL), connective tissue growth factor (CTGF) and serum deprivation response (SDPR), which contribute to the malignant phenotype [101]. Another transcriptional target of ZEB1 is DEAD-Box 56 (DDX56). Once ZEB1 increases the expression of DDX56, it can exert its downstream actions by upregulating the expression of muscle, intestine and stomach expression 1 (MIST1), which in turn negatively regulates Phosphatase and Tensin Homolog (PTEN) to activate AKT signaling, leading to HCC cell proliferation [114]. Besides classical targets, ZEB1 can control the transcription of ncRNAs. For example, ZEB1 activates the expression of the hsa-microRNA-99b/let-7e/microRNA-125a cluster in the HCC cells to promote invasion and advance liver cancer progression [115].

ZEB1 can also influence the metabolism of HCC cells by upregulating the expression of the muscle isoform of PFKM (phosphofructokinase), a rate-limiting enzyme in glycolysis, which stimulates glycolysis and leads to increased malignant phenotypes through promotion of the Warburg effect [112], a well-known mechanism used by cells undergoing EMT to fuel metabolic needs [116,117]. Similarly, ZEB1 activates the transcription of phosphoglycerate dehydrogenase (PHGDH), which in turn augments the serine synthesis pathway to render the cells more proliferative and invasive and resistant to sorafenib [111]. Activation of the serine biosynthetic pathway is involved in the induction of stemness and chemoresistance across tumor types [118,119]. In HCC cells, PHGDH promotes the translation of mitochondrial DNA-encoded proteins and sustains respiration [120], which helps with meeting the metabolic needs of these cells. This metabolic rewiring has been successfully targeted in other cancers but remains unexplored in HCC [118,119]. Curiously, both PFKM and PHGDH are regulated through non-classic ZEB1 binding sites within their promoter regions [111,112].

Regarding chemoresistance, ZEB1 expression has been shown to increase in HCC cells resistant to oxaliplatin, doxorubicin, sorafenib and bortezomib. These cells are usually enriched in the CD44high/CD24low stem cell population and show augmented expression of Protein kinase C alpha (PKCA) [121]. ZEB1-induced EMT has been linked to resistance to sorafenib [21,46,111], lenvatinib [52] and bortezomib [122], but the exact ZEB1-dependent downstream mechanism has not yet been delineated in these studies.

ZEB1 has also been implicated in the interactions between HCC cells and the TME. ZEB1 interacts with USP22 to coactivate transcriptionally vascular endothelial growth factor A (VEGFA). Then, VEGFA is able to act in a paracrine way to induce cell proliferation, migration and vascular mimicry formation in HCC cells and angiogenesis by acting on endothelial cells [58]. ZEB1 is not only expressed in HCC cells, but also in the TME. Indeed, miR-200b-3p can be encapsulated in exosomes that are taken by macrophages from the TME. In these cells, miR-200b-3p reduces ZEB1 expression, which negatively regulates interleukin 4 (IL4). Thus, increased IL4 levels promote a shift to M2 macrophage phenotype, with increased Pim-1 proto-oncogene, serine/threonine kinase (PIM1) and VEGFA production by the macrophages that act on HCC cells to promote proliferation and metastasis [123].

## 5. Role of ZEB1 in Cholangiocarcinoma (CCA)

Regarding clinical analysis, nuclear ZEB1 expression has been shown to be highly expressed in malignant intrahepatic CCA cells compared to normal non-neoplastic cells in 20–46.1% of the cases (Table 2) [14,110]. According to its role in EMT, ZEB1 expression showed a significant inverse correlation with E-cadherin expression and a positive correlation with vimentin expression [110]. Interestingly, ZEB1 expression has been associated with programmed cell death ligand 1 (PD-L1) expression in CCA [124]. Although this association has been demonstrated in other tumors [125], there is no mechanistic validation yet in CCA. Importantly, ZEB1 expression was associated with aggressive tumor characteristics, including advanced tumor stage, undifferentiated-type histology, lymph node metastasis and vascular invasion (Table 3) [14,111,127]. Furthermore, patients with high ZEB1 expression showed significantly reduced overall survival rates (Table 2) [111,127].

**Figure 5:**
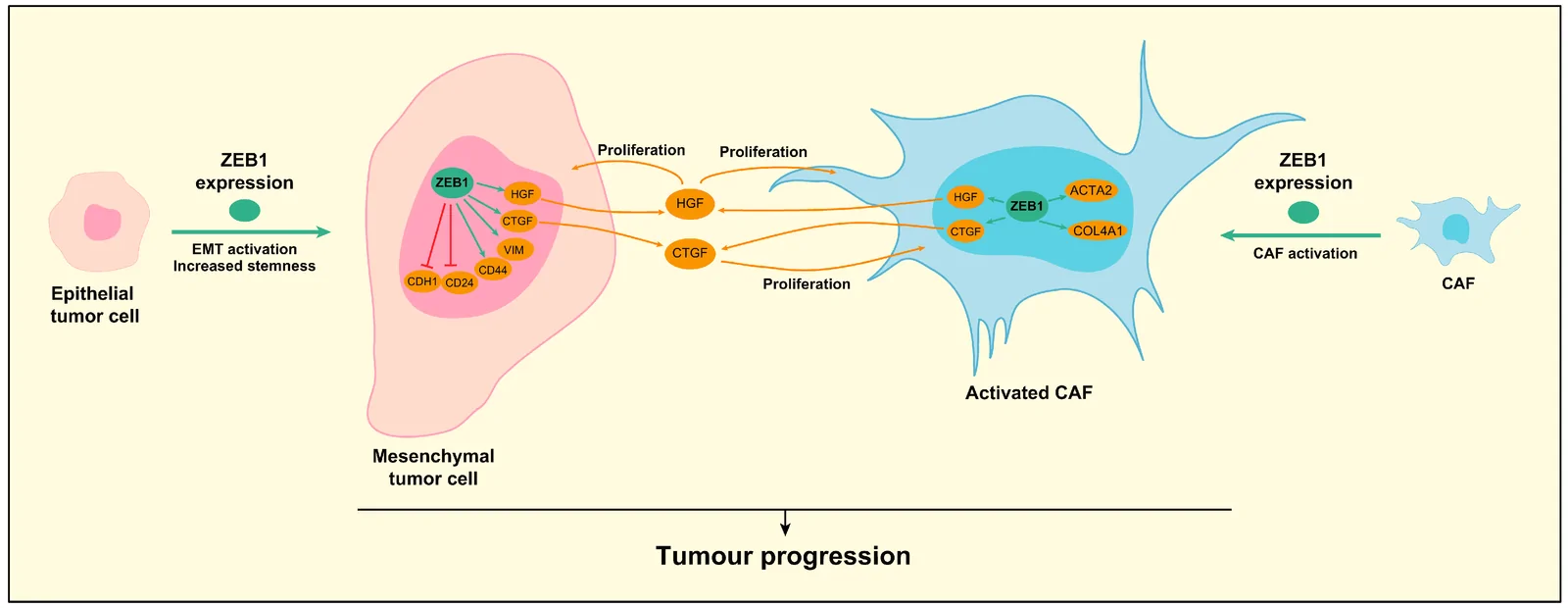
Schematic representation of the mechanisms governing CCA tumor progression that are regulated by ZEB1 in tumor cells and the tumor microenvironment. *ACTA2*, Actin alpha 2, smooth muscle; *CDH1*, E-cadherin; *COL4A1*, collagen 4A1; CTGF, connective tissue growth factor; HGF, hepatocyte growth factor; VIM, vimentin; ZEB1, zinc finger E-box binding homeobox 1.

In vitro, ZEB1 has been shown to promote the proliferation, colony formation, sphere formation and migration of CCA cells and markedly enhance tumor progression in vivo [14,126]. ZEB1 can promote these features by several mechanisms, summarized in Figure 5. On one side, ZEB1 promotes cell migration through the activation of EMT, which leads to downregulation of epithelial markers, such as E-cadherin, and upregulation of mesenchymal markers, such as vimentin [14]. Among the molecular mechanisms related to ZEB1-induced EMT, the feedback loop with miR-200 has been identified in CCA cells [127]. On the other side, ZEB1 increases the stemness of CCA cells by altering the balance of the stem cell markers [14]. ZEB1 upregulates the expression of CD44 [14] (and its alternative splicing) [126], while it downregulates CD24 expression [14], generating a population of cells with high stemness [128,129]. Accordingly, subcutaneous implantation of CCA cells with ZEB1 overexpression produced bigger tumors in nude mice [14]. Curiously, tumors formed by ZEB1 overexpressing cells showed a more abundant stroma than their counterparts. The underlying mechanism involved the direct regulation by ZEB1 of CTGF in CCA cells, which in turn activated CAF proliferation, which could be impaired by a neutralizing antibody [14]. Interestingly, ZEB1 expression has also been detected in CAF from CCA and in different hepatic stellate cell lines. Downregulation of ZEB1 in these cells produced a downregulation of typical activation markers, such as Actin alpha 2, smooth muscle (*ACTA2*) and Collagen 4A1 (*COL4A1*). ZEB1 expression in the fibroblasts also impacted CCA cell growth [14]. Indeed, conditioned media from fibroblasts with high ZEB1 expression induced CCA cell growth and activation of the intracellular signaling pathways, including the signal transducer and activator of transcription 3 (STAT3), AKT and ERK. Further analysis showed that ZEB1 upregulated the expression of hepatocyte growth factor (HGF) in liver fibroblasts that induced proliferation of both CCA cells and fibroblasts [14]. These data underscore the role of ZEB1 in regulating tumor cell-CAF crosstalk as promoting tumor dedifferentiation and CAF activation, resulting in tumor progression.

## 6. Future Perspectives and Therapeutic Opportunities

Liver cancer is one of the leading causes of cancer-related mortality [3], mainly due to the fact that at diagnosis, most patients already present advanced stages of the disease and distant metastasis [1,2]. Consequently, understanding the process of EMT, a key driver of the metastatic cascade, is essential to tackle this important issue. In this context, an increasing number of publications is showing that ZEB1 expression is increased in both HCC and CCA, and it promotes the aggressiveness of these tumors by regulating multiple biological processes related to cell plasticity, including not only EMT, but stemness, therapy resistance and several aspects of the TME development. Thus, understanding the complex regulatory network supervised by ZEB1 may have important clinical and therapeutic implications.

The main issue with exploiting ZEB1’s therapeutic potential is that ZEB1-specific inhibitors are still to be developed. Nevertheless, some indirect approximations have been taken in liver cancer preclinical studies to inhibit ZEB1-related functions (Table 4).

### 6.1. Chemical Compounds

Farrerol, a bioactive constituent of Rhododendron, is able to suppress TGF-β-mediated migration and invasiveness in HCC cells by inhibiting SMAD2/3 phosphorylation (Figure 2). This impaired phosphorylation led to downregulation of different EMT regulators, including ZEB1 [130]. Similarly, cinobufotalin, a bufadienolide isolated from toad venom, downregulates β-catenin-dependent EMT, which is in part mediated by ZEB1 (Figure 2) [131]. Another report shows that salinomycin, an antibacterial and coccidiostat ionophore, activates FOXO3a, which acts as a negative regulator of the β-catenin, with similar results, reducing ZEB1 expression and EMT in HCC cells (Figure 2). Interestingly, this mechanism mediated by salinomycin is able to revert resistance to doxorubicin in these cells [28]. In CCA, dihydromyricetin (DMY), the main bioactive compound isolated from *Ampelopsis grossedentata*, significantly reduces proliferation and EMT in tumor cells, through a mechanism involving miR-455-3p upregulation, which in turn targets and inhibits ZEB1 expression [88]. Also in CCA, maraviroc, a CCR5 inhibitor, has been used to impair CCL5-regulated functions upstream of ZEB1 [132]. Additionally, post-translational modifications such as O-GlcNAcylation can be exploited to impair ZEB1 functions. Artesunate, a key antimalarial drug, which originated from the traditional Chinese medicinal plant *Artemisia annua*, promotes OGA function, which is in charge of removing the O-linked N-acetylglucosamine modification at ser 670, reducing ZEB1 protein stability (Figure 3) [56]. Besides targeting ZEB1 expression, PKC inhibitors have been used to inhibit ZEB1-regulated downstream functions that are mediated by PKCA in HCC cells [121]. The main problem of these approaches is that they do not specifically target ZEB1.

### 6.2. Neutralizing Antibodies

Neutralizing antibodies against soluble signaling molecules is another regulatory mechanism that can be exploited against ZEB1. For example, neutralizing antibodies against CCL5, produced by MSC or CAF, have been used to inhibit CCL5-regulated processes, including ZEB1 expression [132].

### 6.3. miRNAs

The potential of miRNAs has been assayed in HCC in a study where miR-708 was used therapeutically to target and downregulate ZEB1 expression [90]. The most accurate therapeutic option available is probably miRNA-based therapy targeting ZEB1 expression. Nevertheless, it is necessary to ensure the specificity of the miRNAs for ZEB1 and to further develop the delivery methods and the stability of the therapeutic agents.

## 7. Conclusions

In conclusion, ZEB1 is a key regulator of transcriptional plasticity in liver cancer, allowing the cells to adapt to challenges from environmental and therapeutic pressures through the activation of EMT, stemness and chemoresistance programs, which contribute to the aggressivity of the tumors and poorer prognosis of liver cancer patients. Future research should aim to untangle the complexity of the ZEB1-regulated network to identify potential new therapeutic targets that could be integrated with current therapies to improve the evolving treatment landscape of liver cancer patients.

## Figures and Tables

**Table 2:** Clinical relevance of ZEB1 in the survival of patients with liver cancer.

Tumor	Sample Size	Detection Method	Cut Off	% ZEB1 Positive/ High Expression	PFS/OS	HR	95%CI	*p*-Value	Ref.
HCC	54	IHC	Expression > 1%	29.6	PFS (MV)	15.5	10.1–20.9	<0.001	[106]
					OS (MV)	9.6	4.34–14.86	<0.001	
HCC	153	IHC	Score > 2	59.4	OS (MV)	1.812	1.060–3.097	0.030	[108]
HCC	110	WB	-	65.4	PFS (MV)	1.814	1.002–3.284	0.048	[109]
					OS (MV)	2.222	1.097–4.503	0.027	
HCC	108	IHC	-	23.0	OS (MV)	1.45	1.02–2.00	0.037	[13]
					OS (UV)	1.20	0.83–1.71	0.320	
CCA	102	IHC	Score 0–300	46.1	OS (UV)	0.911	0.493–1.686	0.027	[110]

CCA, cholangiocarcinoma; CI, confidence interval; HCC, hepatocellular carcinoma; HR, hazard ratio; IHC, immunohistochemistry; MV, multivariate; OS, overall survival, PFS; progression free survival; UV, univariate; WB, Western blot.

**Table 3:** Association of ZEB1 expression with clinicopathological parameters in patients with liver cancer.

Tumor	Parameter	Sample Size	ZEB1 Negative	ZEB1 Positive	*p*-Value	Ref.
HCC	Grade	54	38	16		[106]
	I	12	11	1	0.006	
	II	28	21	7		
	III	14	6	8		
	Staging	54	38	16		
	I	9	8	1	0.001	
	II	20	19	1		
	III	16	7	9		
	IV	9	4	5		
	Vascular Invasion	54	38	16		
	No	21	19	2	0.01	
	Yes	33	19	14		
	Number of metastases	54	38	16		
	Uni-focal	28	26	2	0.001	
	Multi-focal	26	12	14		
	Child pough class	54	38	16		
	A	19	18	1	0.003	
	B	28	17	11		
	C	7	3	4		
HCC	Number of tumors	153	62	91		[107]
	1	82	58	24	<0.001	
	>1	71	4	67		
	Grade	153	62	91		
	Well + moderate	77	44	33	<0.001	
	Poor	76	18	58		
	Staging	153	62	91		
	I	52	44	8	<0.001	
	II	64	7	57		
	III	42	11	20		
	IV	6	0	6		
HCC	Staging	80	38	42		[108]
	I–II	43	14	29	0.04	
	III–IV	37	24	13		
HCC	Staging					[109]
	I	12	8	4	0.010	
	II	30	13	17		
	III–IV	68	17	51		
	Tumor size					
	<5	37	18	19	0.027	
	>5	73	20	53		
	Intrahepatic metastases					
	Absent	82	33	49	0.031	
	Present	28	5	23		
	Vascular invasion					
	Absent	47	23	24	0.006	
	Present	63	15	48		
	Early recurrence					
	Yes	34	6	28	0.013	
	No	76	32	44		
HCC	Vascular invasion	108				[13]
	Absent	70	10	60	0.016	
	Present	38	13	25		
	Staging	108				
	I + II + III	88	15	73	0.023	
	IV	20	8	12		
CCA	Sex	102	55	47		[110]
	Men	63	29	34	0.042	
	Women	39	26	13		
	Age (years)	102	55	47		
	<64	51	20	31	0.003	
	>64	51	35	16		
	Staging	102	55	47		
	I + II + III	59	37	22	0.037	
	IV	43	18	25		
	Differentiation	102	55	47		
	Well	20	16	4	0.017	
	Moderately	58	30	48		
	Poorly	24	9	15		
	Lymph node metastasis	102	55	47		
	Absent	70	43	27	0.024	
	Present	32	12	20		
	Portal vein invasion	102	55	47		
	Absent	59	37	22	0.037	
	Present	43	18	25		
CCA	Grade	45	35	10		[14]
	I	17	15	2	<0.05	
	II	18	15	3		
	III	10	5	5		
	Vascular Invasion	45	35	10		
	Absent	13	13	0	<0.05	
	Present	32	22	10		

CCA, cholangiocarcinoma, HCC, hepatocellular carcinoma.

**Table 4:** Therapeutic strategies against ZEB1 regulated functions in liver cancer.

Tumor	Strategy	Molecule	Target	Ref.
HCC	Chemical compound	Farrerol	SMAD2/3	[130]
HCC	Chemical compound	Cinobufotalin	β-catenin	[131]
HCC	Chemical compound	Salinomycin	FOXO3a	[28]
CCA	Chemical compound	Dihydromyricetin	miR-455-3p	[88]
HCC	Chemical compound	Artesunate	O-GlcNAcase	[56]
HCC	Chemical compound	PKC inhibitors	PKCA	[121]
CCA	Chemical compound	Maraviroc	CCR5	[132]
CCA	Neutralizing antibody	-	CCL5	[132]
HCC	miRNA	miR-708	ZEB1	[90]

CCA, cholangiocarcinoma; CCL5, C-C motif chemokine ligand 5; CCR5, C-C motif chemokine ligand 5 receptor; FOXO3a, forkhead box O3; HCC, hepatocellular carcinoma; PKCA, Protein kinase C alpha; ZEB1, zinc finger E-box binding homeobox 1.

## Data Availability

No new data were created or analyzed in this study. Data sharing is not applicable to this article.

## Abbreviations

The following abbreviations are used in this manuscript:

ABCA8	ATP binding cassette subfamily A member 8
ACTA2	Actin alpha 2, smooth muscle
ADM	Adrenomedullin
AKT	AKT serine/threonine kinase
AP-2γ	Activator Protein-2 gamma
AXL	AXL receptor tyrosine kinase
CAF	Cancer-associated fibroblasts
CAPZA1	F-actin-capping protein subunit alpha-1
CBD	P300-P/CBP-associated factor (P/CAF) binding domain
CBX6	Chromobox 6
CCA	Cholangiocarcinoma
CCL5	C-C motif chemokine ligand 5
CCR5	C-C motif chemokine ligand 5 Receptor
CID	CtBP interaction domain
circRNA	Circular RNA
COL4A1	Collagen 4A1
CTGF	Connective tissue growth factor
CUL4A	Cullin 4A
DDX56	DEAD-Box 56
DEN	Diethylnitrosamine
DMY	Dihydromyricetin
EGF	Epidermal growth factor
EGR1	Early growth response 1
eEF-1γ	Eukaryotic translation elongation factor 1 gamma
EMT	Epithelial–mesenchymal Transition
EMT-TF	EMT-inducing transcription factor
ERK	Extracellular signal-regulated kinase
FBXW7	F-Box and WD repeat domain containing 7
FBXW8	F-box and WD repeat domain containing 8
FGF	Fibroblast growth factor
FGG	Fibrinogen gamma chain
FOXO3a	Forkhead box O3
GINS1	GINS complex subunit 1
GSK3β	Glycogen synthase kinase 3 beta
HCC	Hepatocellular carcinoma
HDAC	Histone deacetylase
HGF	Hepatocyte growth factor
HIF1α	Hypoxia-inducible factor 1 alpha
HIPK2	Homeodomain Interacting Protein Kinase 2
HMGB1	High mobility group box 1
HMGB2	High mobility group box 1
HN1L	Hematological and neurological expressed 1-like
IGFBP2	Insulin like growth factor binding protein 2
IL4	Interleukin 4
LKB1	Liver kinase B1
lncRNA	long non-coding RNA
m6A	N6-methyladenosine
MAPK	Mitogen-activated protein kinase
MAZ	MYC associated zinc finger protein
METTL3	Methyltransferase-like protein 3
METTL13	Methyltransferase 13
miRNA	MicroRNA
MIST	Muscle, intestine and stomach expression 1
MMP2	Matrix metallopeptidase 2
MSC	Mesenchymal stem cells
mTOR	Mechanistic target of rapamycin kinase
ncRNAs	Non-coding RNAs
NCSTN	Nicastrin
NFκB	Nuclear factor kappa B
NONO	Non-POU domain containing octamer binding
OTUB2	OTU deubiquitinase, ubiquitin aldehyde binding 2
PDHA-1	Pyruvate dehydrogenase E1 subunit alpha 1
PFKM	Phosphofructokinase-1, muscle
PHGDH	Phosphoglycerate dehydrogenase
PIM1	Pim-1 proto-oncogene, serine/threonine kinase
PKCA	Protein kinase C alpha
PKM2	Pyruvate kinase M2
POU2F1	POU class 2 homeobox 1
PPM1G	Protein phosphatase 1G
PPT1	Palmitoyl-Protein Thioesterase 1
PTEN	Phosphatase and tensin homolog
PTK7	Tyrosine-protein kinase-like 7
PTP4A1	Protein tyrosine phosphatase type 4A1
RBMY	RNA-binding motif, Y chromosome
SDPR	Serum deprivation response
SET	Suppressor of variegation, enhancer of zeste, trithorax
SID	Smad interaction domain
SMYD3	MYND (Myeloid-Nervy-DEAF1) domain containing 3
SNAI1/2	Snail family transcriptional repressor 1/2
SORT1	Sortilin 1
SP1	Specificity protein 1
SSEA3	Stage-specific embryonic antigen 3
STAT3	Signal Transducer and activator of transcription 3
TBL1X	Transducin beta like 1 X-linked
TCF	T-cell factor
TF	Transcription factor
TGF-β	Transforming growth factor-beta
THRSP	Thyroid Hormone Responsive Protein
TME	Tumor microenvironment
TPA	O-tetradecanoyl-phorbol 13-acetate
TRAF6	TNF receptor associated factor 6
TRIM26	Tripartite motif containing 26
TRPV1	Transient receptor potential cation channel subfamily V member 1
TWIST1/2	Twist family bHLH transcription factor 1/2
TXNDC12	Thioredoxin domain containing 12
UPAT	Ubiquitin-like plant homeodomain (PHD) and really interesting new gene (RING) finger domain-containing protein 1 (UHRF1) protein associated transcript
USP22	Ubiquitin specific peptidase 22
USP39	Ubiquitin specific peptidase 39
VEGFA	Vascular endothelial growth factor A
Wnt	Wingless/integrated
YAP	Yes-associated protein
YTHDF3	YTH domain-containing family protein 3
ZEB1/2	Zinc finger E-box-binding homeobox 1/2
ZIP4	Zrt- and irt-like protein 4
