# Correction: Gonzalez-Sanchez et al. Epithelial to Mesenchymal Transition Transcriptional Regulator ZEB1 in Liver Cancer: Oncogenic Roles and Therapeutic Potential. *Int. J. Mol. Sci.* 2025, *26*, 11135

**DOI:** 10.3390/ijms27156676

**Published:** 2026-07-27

**Authors:** Ester Gonzalez-Sanchez, Carlos Andres Roldan-Hernandez, Ana Martin-Ramirez, Lucia Garcia-Collado, Laura Fouassier, Javier Vaquero

**Affiliations:** 1 HepatoBiliary Tumours Lab, Centro de Investigación del Cáncer and Instituto de Biología Molecular y Celular del Cáncer, CSIC-Universidad de Salamanca, 37007 Salamanca, Spain; e.gonzalezsan@usal.es (E.G.-S.); croldanh@usal.es (C.A.R.-H.); anamr02@usal.es (A.M.-R.); luciiiacollado@usal.es (L.G.-C.); 2 Department of Physiology and Pharmacology, University of Salamanca, 37007 Salamanca, Spain; 3 National Biomedical Research Institute on Liver and Gastrointestinal Diseases (CIBERehd), Instituto de Salud Carlos III, 28029 Madrid, Spain; 4 Université Paris Cité, NABI, CNRS UMR8175, Inserm U1334, F-75006 Paris, France; laura.fouassier@inserm.fr

The following reference [84] has been retracted and has to be removed from the original publication [1]:

84. Gu, Y.; Zhu, Z.; Pei, H.; Xu, D.; Jiang, Y.; Zhang, L.; Xiao, L. Long non-coding RNA NNT-AS1 promotes cholangiocarcinoma cells proliferation and epithelial-to-mesenchymal transition through down-regulating miR-203. *Aging* **2020**, *12*, 2333–2346.

The scientific content of [84] could not be replaced with any other publication. Therefore, the authors decided to remove reference [84]. Also, the authors have now revised tables and text and deleted the relevant text passages that originally cited this article. With this correction, the order of some references has been adjusted accordingly.

The authors state that the scientific conclusions are unaffected. This correction was approved by the Academic Editor. The original publication has also been updated.
